# A Textile-Based Wearable Sensing Device Designed for Monitoring the Flexion Angle of Elbow and Knee Movements

**DOI:** 10.3390/s140304050

**Published:** 2014-02-26

**Authors:** Tien-Wei Shyr, Jing-Wen Shie, Chang-Han Jiang, Jung-Jen Li

**Affiliations:** 1 Department of Fiber and Composite Materials, Feng Chia University, 100 Wenhwa Road, Seatwen, Taichung 40724, Taiwan; E-Mails: doublemoonclan@gmail.com (C.-H.J.); dobiafish@gmail.com (J.-J.L.); 2 Taiwan Textile Research Institute, 20 Kejia Road, Douliu City, Yunlin 64057, Taiwan; E-Mail: jwshie@gmail.com

**Keywords:** wearable gesture-sensing device, textile strain sensor, elastic conductive webbing, flexion angle monitor, electronic textiles

## Abstract

In this work a wearable gesture sensing device consisting of a textile strain sensor, using elastic conductive webbing, was designed for monitoring the flexion angle of elbow and knee movements. The elastic conductive webbing shows a linear response of resistance to the flexion angle. The wearable gesture sensing device was calibrated and then the flexion angle-resistance equation was established using an assembled gesture sensing apparatus with a variable resistor and a protractor. The proposed device successfully monitored the flexion angle during elbow and knee movements.

## 1. Introduction

Measuring human movements is important before rehabilitation, training or exercises can start. Motion capture systems that use cameras, accelerometers, and flexible electrogoniometers have been studied extensively [1–5]. Although motion capture systems are capable of accurate measurements, they are inconvenient to use. Either the measurements must be set based on the characteristics of the person, or the axis of the coordination system has to be reset [2]. Moreover, the construction of the system requires fairly substantial and heavy equipment. The main drawbacks of the motion capture systems available in today's market are their weight and rigidity. In particular, conventional sensors often require the use of complex and uncomfortable mechanical plug-ins in order to position the sensors on garments [6].

Textile solutions are well suited for constructing a sensing system that is comfortable for the wearer. Several studies have been carried out to show that a textile sensor is a practical and wearable electric device [6–9]. Electronic textiles (e-textiles) are used in the entertainment industry and the fashion industry, as well as for communication, sensing and monitoring, even for position location [10–15]. Due to the rigidity of an alloy strain gauge, the maximum static strain level is limited to low stress measurements. Fatigue is another problem of alloys when making dynamic measurements because of their poor repeatability [16]. Therefore one particularly interesting application for e-textiles is their use as strain-resistance sensors.

Abdessalem reported that plated plain knitted fabric using Lycra yarns exhibited serious tensile hysteresis [17]. The incomplete recovery of a knitted fabric is mainly due to the slippage of the fibers in spun yarn and/or the permanent extension of the fibers. The permanent extension of the fibers depends on the viscoelasticity of the fiber in used. Mattmann developed a strain sensor using a mixture of thermoplastic elastomer and carbon black particles [18]. The strain sensor was proven to have a linear response of resistance to the strain, but with a small electrical hysteresis. The use of carbon coated yarns wrapped with elastic yarn as a strain sensor was studied by Huang [19,20]. However the strain-resistance relationship of the sensor was found to be non-linear, which was mainly due to the irregular characteristic of the yarn structure.

In our previous study we used fabricated elastic conductive webbing using carbon coated fibers and elastic fibers as a strain-resistance sensor [21]. It showed to have high resistance sensitivity, low tensile hysteresis, as well as high linearity and repeatability of the relationship between strain and resistance without resistance hysteresis. In the present study we developed a wearable gesture-sensing device consisting of a strain-resistance textile sensor, based on elastic conductive webbing, for monitoring the flexion angle during elbow and knee movements. We established the flexion angle-resistance equation of the strain-resistance textile sensor and then evaluated the performance of the wearable sensing device for monitoring the flexion angle during elbow and knee movements.

## 2. Experimental Section

### 2.1. Materials

We used elastic conductive webbing made with conductive yarns and elastic yarns in this study. Polyamide fiber coated with carbon particles (PAC fiber) and having a diameter of 50 μm was used as the conductive fiber. Fifteen PAC fibers were twisted with a polyester yarn at a rate of 80 twists per meter to form a conductive yarn with a diameter of 420 μm. Then a Lycra fiber was cross-wrapped over two polyester yarns to form an elastic yarn with a diameter of 800 μm. The tensile properties and the resistance of this elastic and conductive yarn were reported in our previous study [21].

**Figure 1.**
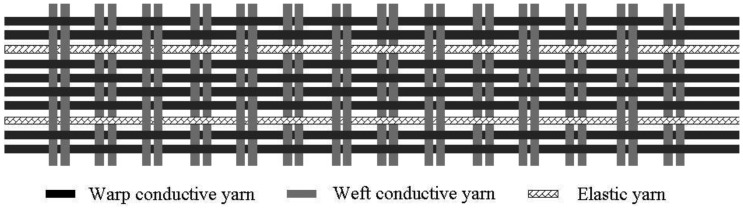
The elastic conductive webbing with a plain structure.

The elastic conductive webbing has a plain structure, 8 mm wide by 2 mm thick. The warp is made up of 32 conductive yarns and five elastic yarns, and the weft by one conductive yarn (see Figure 1). The feed ratio of the conductive yarn in the warp direction was 280%. The elastic yarns were positioned between the conductive yarns as stuffer yarns in warp direction.

### 2.2. The Assembled Gesture Sensing Apparatus and Calibration

**Figure 2.**
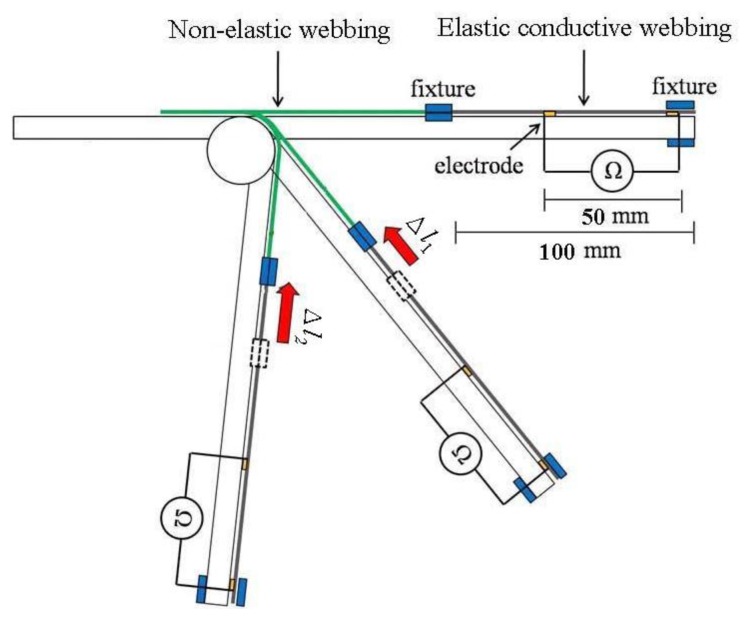
Schematic of the assembled gesture sensing apparatus for measuring the flexion angle-resistance.

A gesture sensing apparatus with a protractor was assembled for measuring the flexion angle-resistance (Figure 2). The protractor was set on the bearing. The distance between the electrodes was fixed at 50 mm, and the measured length of elastic conductive webbing was 100 mm. In order to obtain a stable resistance when measuring the flexion angle-resistance, the elastic conductive webbing was placed on a flat plane on the apparatus. A non-elastic webbing was connected with the elastic conductive webbing to keep the elastic conductive webbing moving in the flat plane and to pull on the elastic conductive webbing during the flexion-recovery movement. The flexion angle of the gesture sensing apparatus was recorded using a protractor and the change in resistance of the elastic conductive webbing between two electrodes was measured during the flexion-recovery movement. The relationship between the flexion angle and the resistance of the elastic conductive webbing in the flexion-recovery cycles can be established using this assembled gesture sensing apparatus with a protractor.

**Figure 3.**
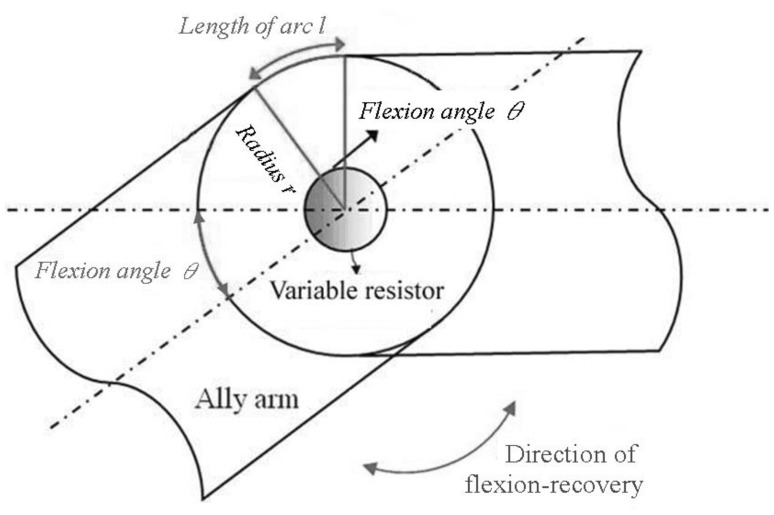
The bearing diagram of the gesture sensing apparatus using a variable resistor.

**Figure 4.**
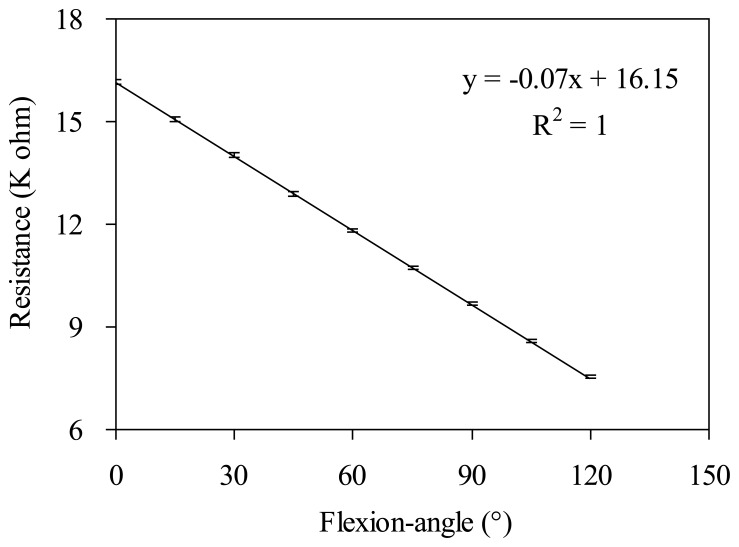
The relationship of the flexion angle and the resistance of the gesture sensing apparatus using a variable resistor and a protractor (The data are averaged from ten flexion-recovery cycles measurements).

A variable resistor and a protractor were used to calibrate the relationship between the flexion angle and the resistance of the assembled gesture sensing apparatus (see Figure 3). Here the flexion angle is proportional to the length of the flexion arc, *l* = 2*πr* × *θ*/360°, where *l* is the arc length, *r* is the radius, and *θ* is the flexion angle. The resistance and flexion angle of the gesture sensing apparatus using a variable resistor and a protractor were recorded for ten flexion-recovery cycles measurements, respectively. The results are shown in Figure 4. It can be seen that the resistance is proportional to the flexion angle. The linear regression equation of the flexion angle to the resistance of the variable resistor on the gesture sensing apparatus is: *y* = −0.07*x* + 16.15 with *R*^2^ = 1, where *x* is the flexion angle, *y* is the resistance, and *R*^2^ is the coefficient of determination.

### 2.3. Design of Textile Strain Sensor

A textile strain sensor was used on the wearable gesture sensing device to determine the resistance in response to the strain. The textile strain sensor consisted of the elastic conductive webbing, two electrodes, two wires, a substrate, and a non-elastic webbing. The substrate was a thin board coated with polytetrafluoroethylene in order to reduce the abrasion between the webbing and the substrate during dynamic movement. The non-elastic webbing was connected with the elastic conductive webbing in order to pull the elastic conductive webbing during the flexion-recovery movement. A copper tube placed on the substrate was used as one electrode, and a stainless steel yarn was used as the electrode connected with the elastic conductive webbing. The distance between the two electrodes was fixed at 50 mm. The change in resistance of the textile strain sensor during the stretching and recovering movements can thus be measured. Based on the relationship between the flexion angle and the resistance of the elastic conductive webbing in the flexion-recovery cycles, the relationship between the flexion angle and the resistance of the textile strain sensor during the stretching and recovering movements can thus be obtained.

### 2.4. Design of the Wearable Gesture Sensing Device

**Figure 5.**
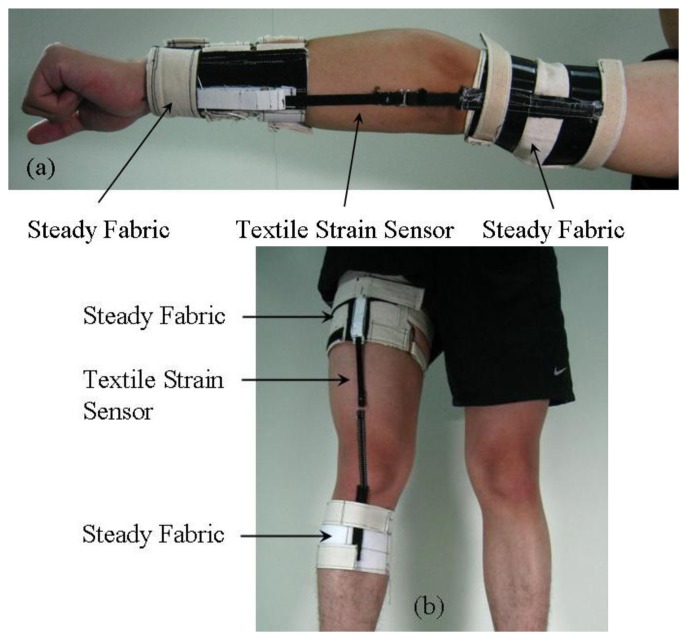
The proposed wearable gesture sensing devices for monitoring the flexion angle during (**a**) elbow and (**b**) knee movements.

The textile strain sensor was placed in the straight position of the limbs for monitoring the flexion angle during elbow or knee movement. In order to ensure the stability of the resistance when measuring the flexion angle-resistance, a woven fabric, referred to as the steady fabric, was used to fix the textile strain sensor. The design of the wearable gesture-sensing devices for measuring the flexion angle during elbow and knee movements is shown in Figure 5.

## 3. Results and Discussion

### 3.1. The Flexion Angle-Resistance of the Elastic Conductive Webbing

**Figure 6.**
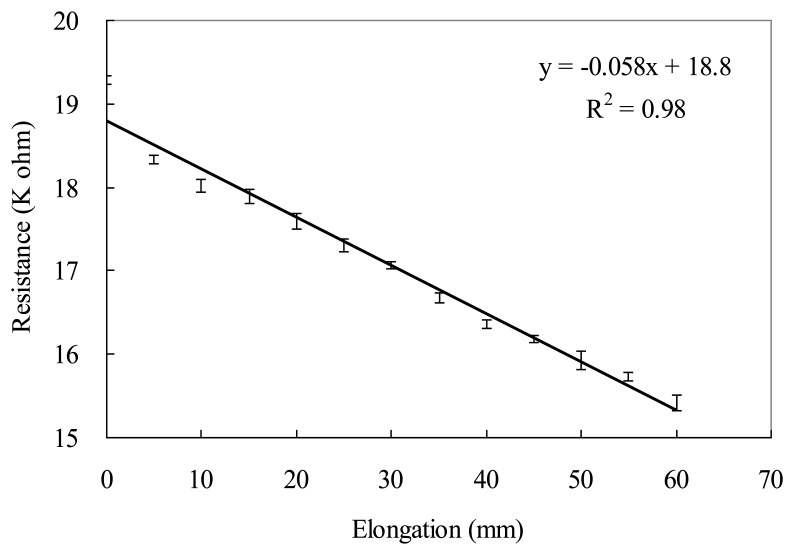
The relationship between elongation and resistance of the elastic conductive webbing (The data are averaged from ten stretch-recovery cycles measurements within 30% strain).

**Figure 7.**
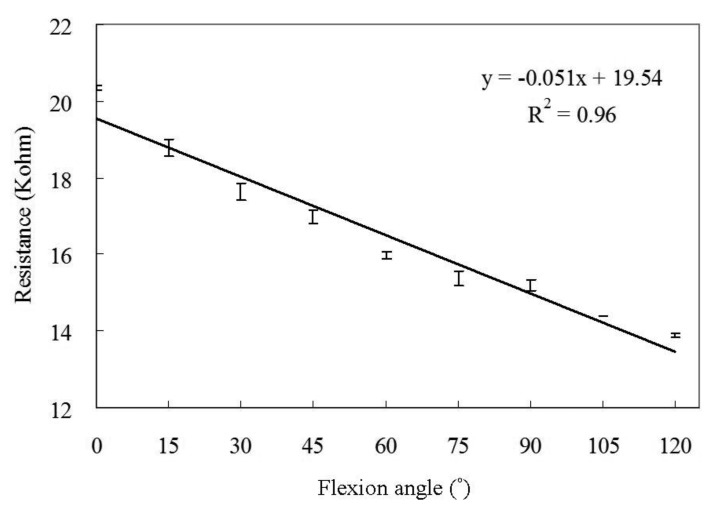
The flexion angle-resistance of the elastic conductive webbing (The data are averaged from ten flexion-recovery cycles measurements).

The resistances to elongation by the elastic conductive webbing during the tensile stretch-recovery cycles within 30% strain are shown in Figure 6. The results show that the resistance of the elastic conductive webbing is proportional to the elongation during the tensile stretch-recovery cycles, in which the coefficient of determination (*R*^2^) of the linear regression curve was 0.98. The flexion angle-resistance of the elastic conductive webbing was measured in ten flexion-recovery cycles within 30% strain using the assembled gesture sensing apparatus with a variable resistor and a protractor. The flexion angle-resistance regression curve of the elastic conductive webbing is shown in Figure 7. The coefficient of determination (*R^2^*) of the linear regression curve is 0.96. This indicates that the elastic conductive webbing has a good linear relationship between flexion angle and resistance during the flexion-recovery cycles within 30% strain.

### 3.2. Developing the Flexion Angle-Resistance Equation of the Wearable Gesture Sensing Device

**Figure 8.**
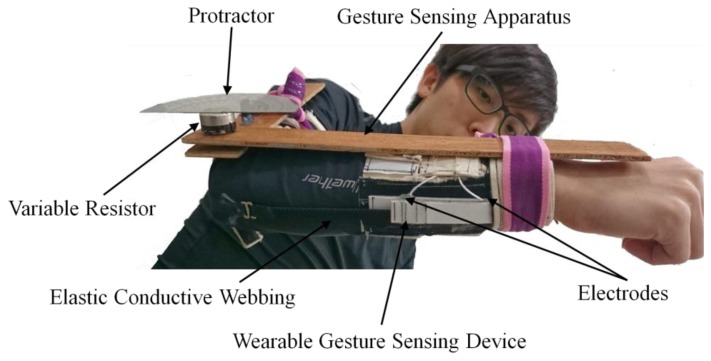
The wearable gesture sensing device and the gesture sensing apparatus with a variable resistor and a protractor on the synchronization process to monitor the flexion angle during elbow movement.

In order to establish the flexion angle-resistance equations of the wearable gesture sensing device for elbow and knee movements, a Data Acquisition/Switch Unit (34972A LXI, Agilent Technologies, Taipei, Taiwan) was used to measure the resistance of textile strain sensor. The wearable gesture sensing device and the gesture sensing apparatus with a variable resistor and a protractor were worn on the elbow or the knee at the same position (Figure 8). Both were synchronously monitoring the flexion angle during elbow or knee movements. The relationship between the flexion angle (°) and resistance (K ohm) of the gesture sensing apparatus with a variable resistor and a protractor was established first. The relationship between flexion angle and resistance of the wearable gesture sensing device was then calibrated using the gesture sensing apparatus with a variable resistor and a protractor. The flexion angle-resistance equations of the wearable gesture sensing device were then established as: *y* = −37*x* + 595 (*R*^2^ = 0.96) and *y* = −19*x* + 280 (*R*^2^ = 0.97) for elbow and knee movements, respectively. Where x is resistance, y is flexion angle, and *R*^2^ is the coefficient of determination.

### 3.3. Evaluating the Wearable Gesture Sensing Device

**Figure 9.**
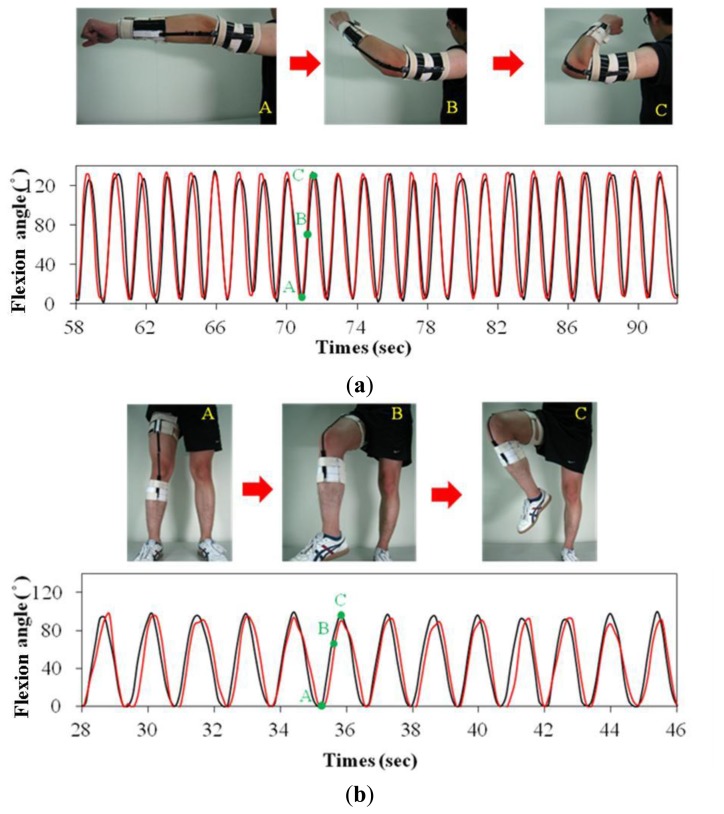
The flexion angle-resistance measurements using the wearable gesture sensing device (red line) and the gesture sensing apparatus with a variable resistor and a protractor (black line) during (**a**) elbow and (**b**) knee movements (Points A, B, and C in the flexion angle-time traces correspond to photos A, B, and C).

The wearable gesture sensing device was applied to monitor the flexion angle during elbow and knee movements. The flexion angle and resistance of the gesture sensing apparatus with a variable resistor and a protractor worn on the same position were synchronously recorded during elbow and knee movements. Figure 9 shows camera snapshots during elbow and knee movements of the wearable gesture sensing device. The resistance of the wearable gesture sensing device during elbow and knee movements was recorded, and the corresponding flexion angle was calculated using the flexion angle-resistance equation of the wearable gesture sensing device (see Figure 9). Compared with the results of the gesture sensing apparatus with a variable resistor and a protractor (Figure 9), it is evident that the results obtained from the wearable gesture sensing device are consistent with those from the gesture sensing apparatus. This suggests that the wearable gesture sensing device is able to monitor the flexion angles during the elbow and knee movements.

## 4. Conclusions

In this work an elastic conductive webbing with a plain structure, consisting of 32 conductive yarns and five elastic yarns in the warp and one conductive yarn in the weft, was used as a textile strain sensor. The resistance of the elastic conductive webbing to the flexion angle had a good linear relationship during the stretch-recovery cycles within 30% strain. This webbing can be used as a flexion angle-resistance sensor for a wearable gesture sensing device. We also designed a textile-based wearable gesture sensing device for monitoring the flexion angle during elbow and knee movements. The flexion angle-resistance equations of the wearable gesture sensing device during elbow and knee movements were calibrated and established using a gesture sensing apparatus with a variable resistor and a protractor. The wearable gesture sensing device was used to monitor the flexion angles during elbow and knee movements. The results showed that the wearable gesture sensing device based on a textile strain sensor for monitoring the flexion angles during elbow and knee movements was successfully implemented. The main advantages of the proposed textile-based wearable gesture sensing device are precision of measurement, wearability, and for the ability of monitoring flexion angles during elbow and knee movements without any discomfort.
